# Migration of the os peroneum: An enigmatic phenomenon explored through radiological perspectives

**DOI:** 10.1016/j.radcr.2023.11.005

**Published:** 2023-11-27

**Authors:** Caleb Bhatnagar, Hongmin Xu, Andrew Pasion, Nida Shaikh, Mallika Shekhar, Emad Allam

**Affiliations:** Department of Radiology and Medical Imaging, Loyola University Chicago and Loyola University Medical Center, 2160 S 1st Ave, Maywood, IL, 60153 USA

**Keywords:** Os peroneum, Migration, Peroneus longus tendon, Foot pain

## Abstract

The os peroneum is an accessory ossicle located along the lateral aspect of the cuboid bone. Its position can serve as an indicator of peroneus longus tendon (PLT) injury. Imaging studies including radiographs and MRI can help detect malposition of the os peroneum and progressive injuries to the PLT and its associated structures. We report a case of a woman with recurrent foot and ankle pain, demonstrating progressive retraction of the os peroneum, implying severe PLT injury which may have ultimately predisposed her to a traumatic fifth metatarsal base fracture. This case highlights the importance of scrutinizing the appearance and position of the os peroneum on radiographs.

## Background

The os peroneum, an accessory ossicle located within the peroneus longus tendon (PLT) along the lateral aspect of the cuboid bone, serves a significant role in foot biomechanics. The PLT, responsible for eversion and plantar flexion of the foot, relies on 3 key anatomical structures for stabilization: the superior and inferior peroneal retinaculum (SPR and IPR), and the long plantar ligament [1,2]. Present in approximately 25% of the population, the os peroneum can exist as a single bone or exhibit multipartite morphology, with pathology potentially leading to lateral foot and ankle pain. Clinically known as painful os peroneum syndrome, this condition encompasses a spectrum of pathologies, including fractures, stress responses, diastasis of a multipartite os peroneum, and injuries to the PLT [3].

Notably, radiographic assessment plays a vital role in diagnosing migration of the os peroneum and associated fractures, which may be associated with concurrent PLT tears. Several studies have described the radiographic findings of os peroneum fractures [1,3–5]. Additionally, the identification of malposition or misalignment of an intact os peroneum on imaging studies can serve as an important indicator of PLT injury and associated damage to its supporting structures.

Radiologists should remain cognizant of these intricate anatomical relationships and pathological conditions when interpreting imaging studies of the foot and ankle, as accurate detection and characterization of os peroneum-related pathologies are important for effective clinical decision-making and patient care.

## Case presentation

**Fig. 1:**
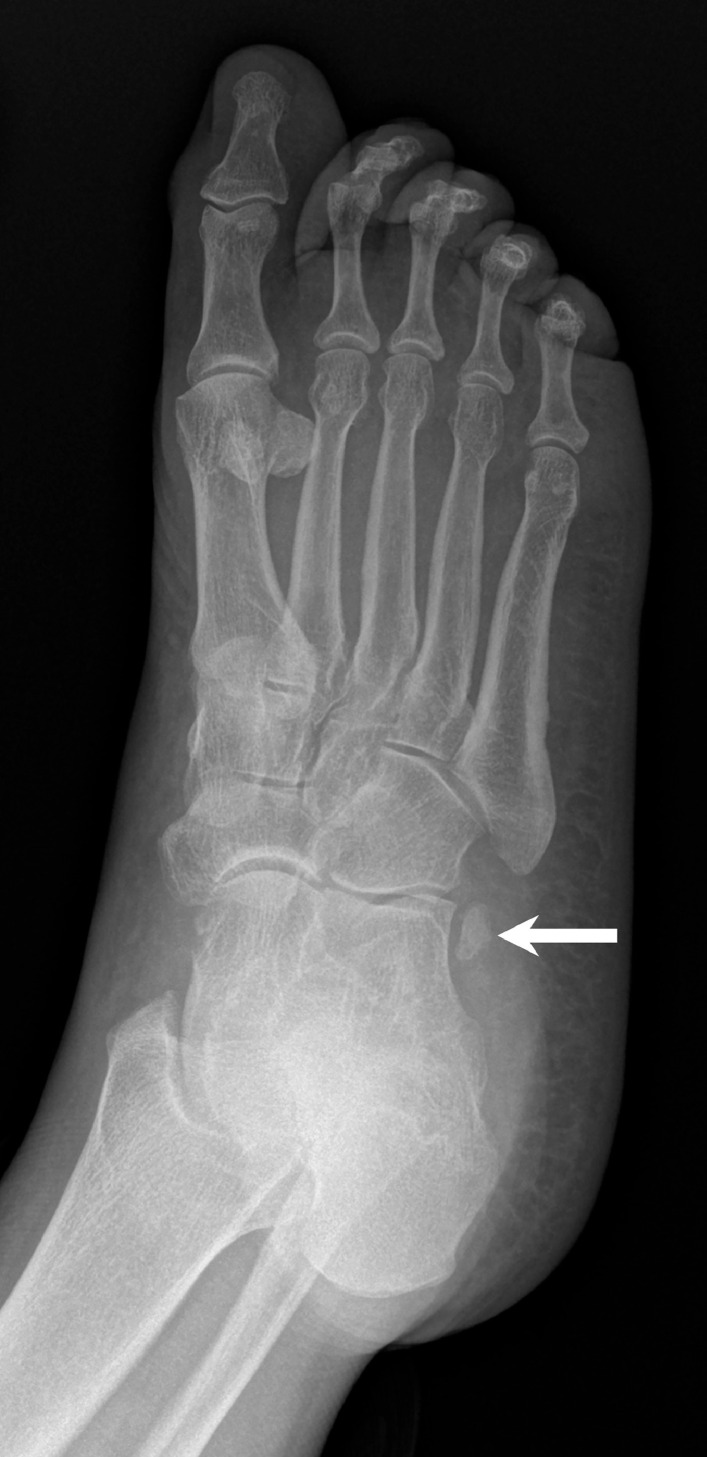
Oblique radiograph of the right foot at the time of initial presentation demonstrates an os peroneum at the expected level of the calcaneocuboid joint (arrow).

We report the case of a 64-year-old woman without relevant medical or surgical history. The patient initially presented with right foot and ankle pain following an injury while walking. Radiographs obtained at the time did not show any acute fracture, and the os peroneum was in its expected position adjacent to the calcaneocuboid joint (Fig. 1).

**Fig. 2:**
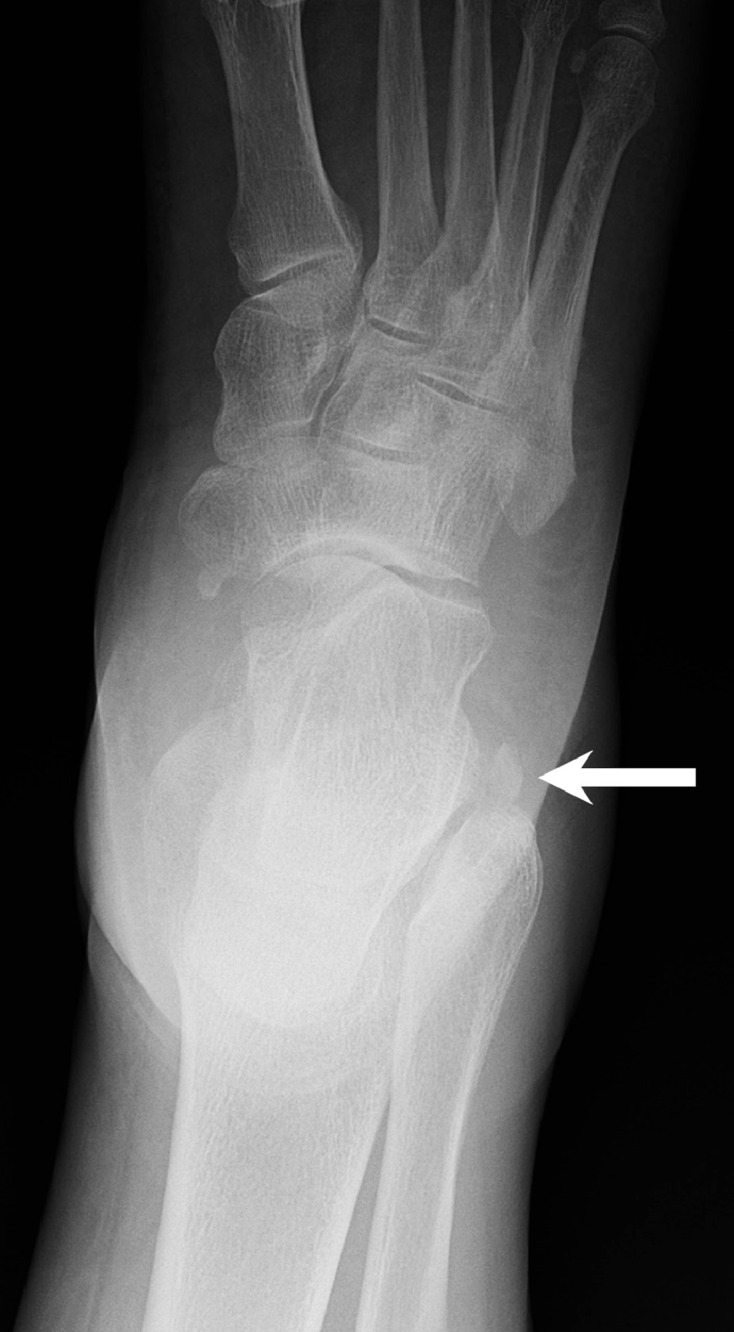
AP radiograph of the right foot 12 days after initial presentation demonstrates proximal migration of the os peroneum to the level of the lateral talus (arrow), suggesting injury to the IPR but an intact SPR.

Twelve days later, the patient returned with increased pain and new radiographs were obtained. The images demonstrated no acute fracture or dislocation. However, the os peroneum had migrated proximally along the lateral aspect of the calcaneus, to the level of the lateral talar process (Fig. 2).

**Fig. 3:**
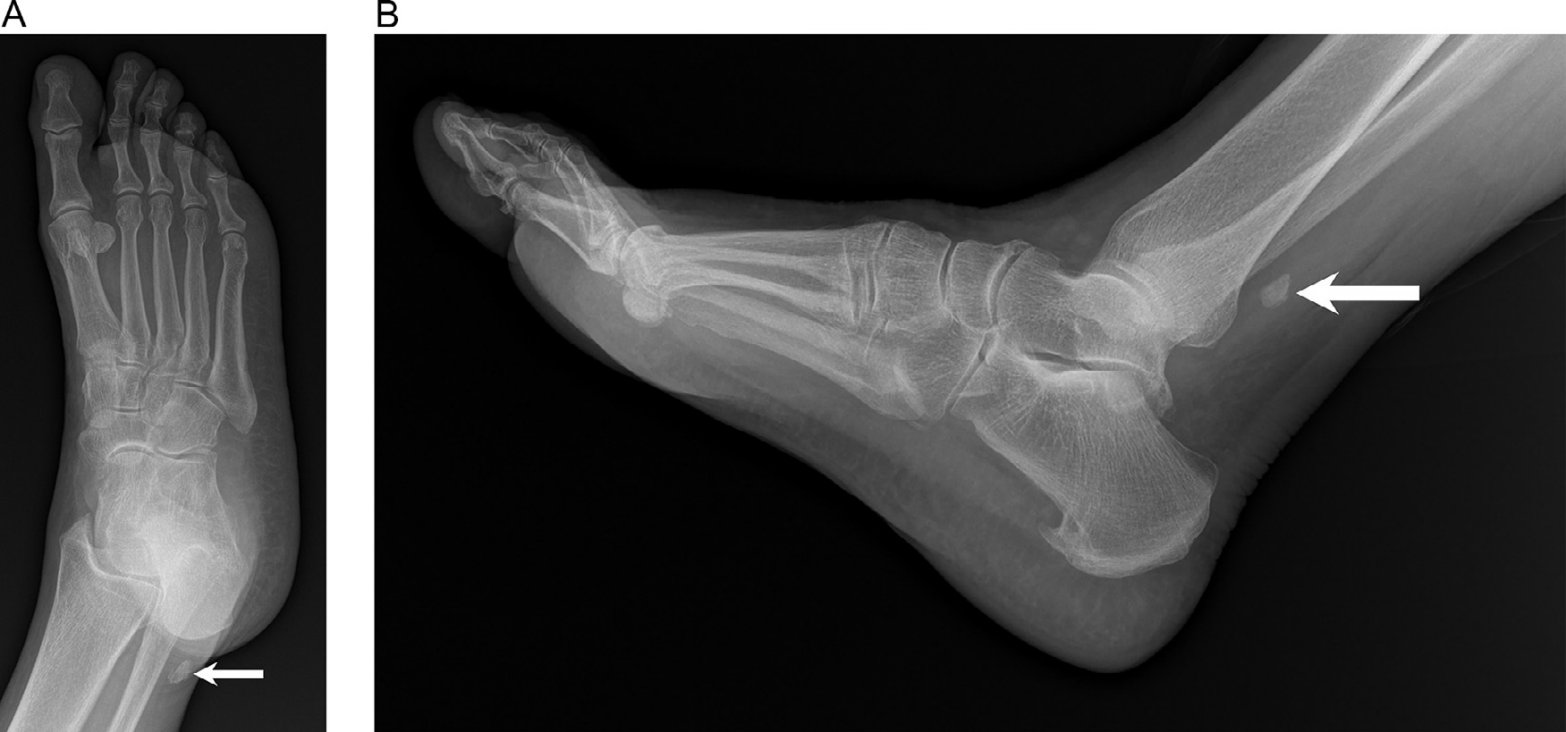
(A) Oblique and (B) lateral radiographs of the right foot 7 months after initial presentation demonstrate further proximal migration of the os peroneum, which is now located posterior to the ankle at the level of distal fibula (arrow). This suggests injury to both the IPR and SPR.

Seven months after the initial presentation, the patient returned with pain in the same location. Radiographs obtained at the time demonstrated no acute fracture or dislocation. However, there was further proximal migration of the os peroneum to the level of the distal fibula, compared to the level of the lateral talus previously (Fig. 3). Injury to the PLT was suggested and conservative management was recommended.

**Fig. 4:**
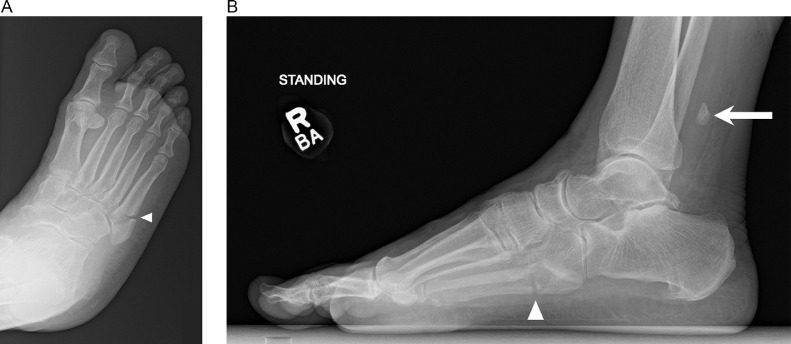
(A) Oblique and (B) lateral radiographs of the right foot 8 months after initial presentation demonstrate a fifth metatarsal base fracture (arrowhead). The os peroneum remains abnormally positioned at level of the distal fibula (arrow).

**Fig. 5:**
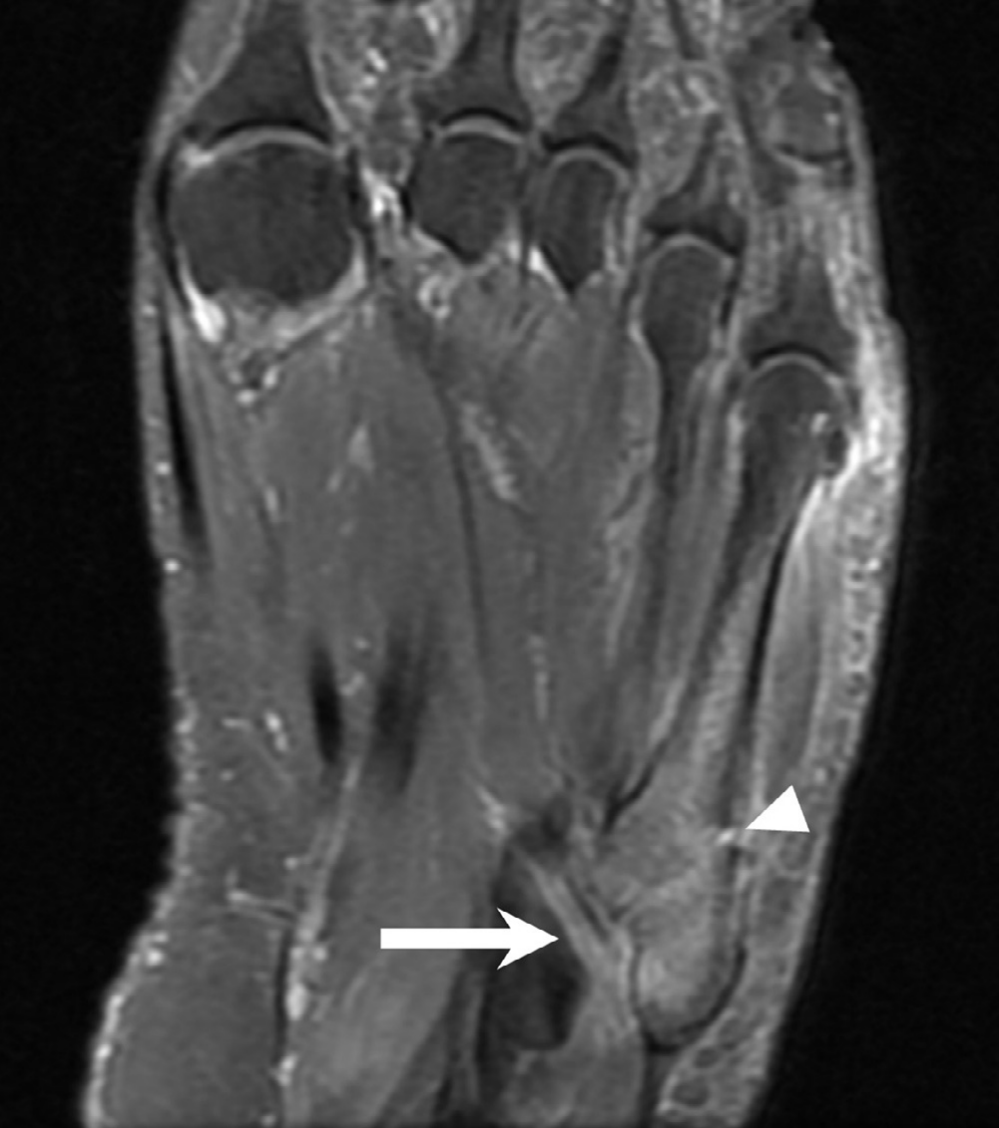
Proton density fat sat MRI sequence of the right foot shows a fracture at the base of the fifth metatarsal (arrowhead) with associated bone marrow edema and peripheral soft tissue edema. Due to known tendon tear and retraction, the PLT is not seen and there is fluid signal in its expected location (arrow).

Eight months after initial presentation, the patient returned again with right foot pain. She had mis-stepped on a curb and rolled her foot and ankle. Radiographs demonstrated a mildly displaced fracture involving the base of the fifth metatarsal; the os peroneum remained at the level of the distal fibula (Fig. 4). Subsequent MRI confirmed a fifth metatarsal base fracture with associated bone marrow edema and peripheral soft tissue edema. The PLT was not seen in its expected position underlying the cuboid, with fluid signal in this location consistent with tendon tear and retraction (Fig. 5). The patient was put in a controlled ankle motion (CAM) boot for immobilization and analgesics were given for pain management.

## Discussion

**Fig. 6:**
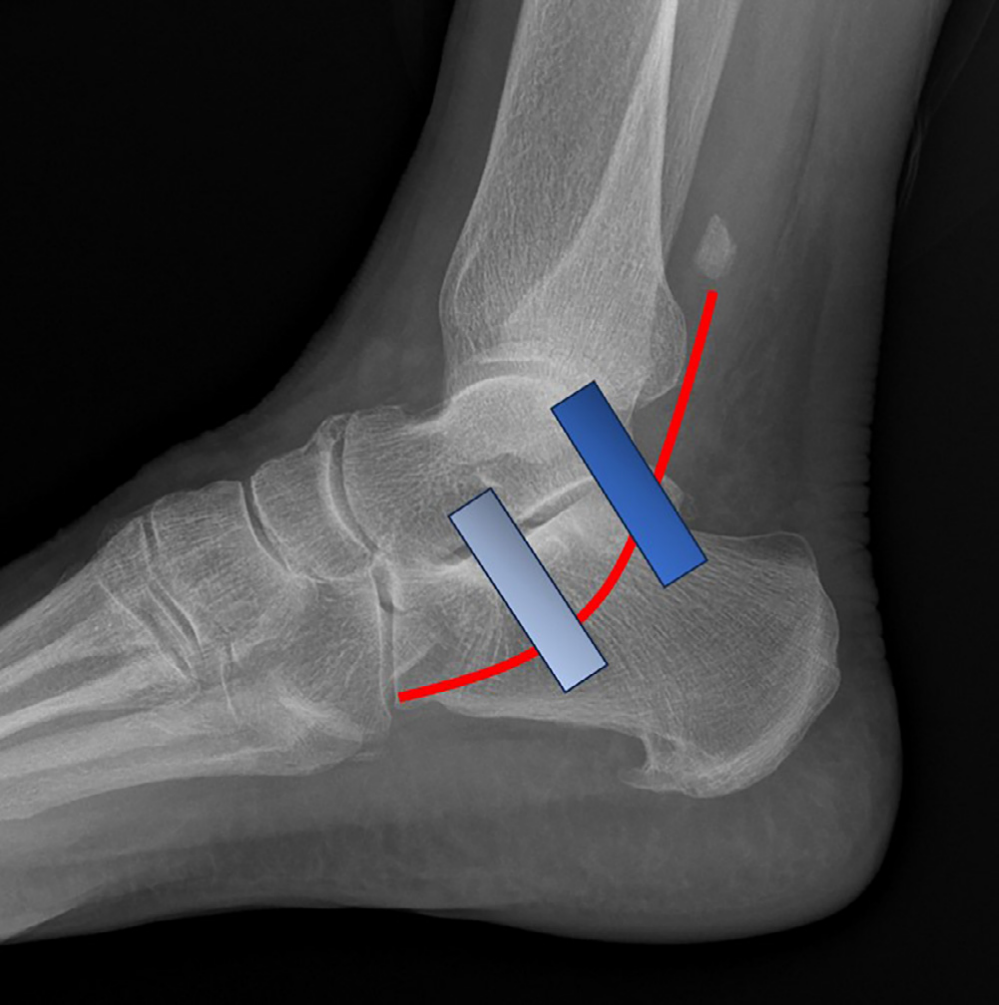
Lateral radiograph of the ankle showing the anticipated migration of the os peroneum within a retracted PLT (red curve). The location of the IPR is shown in light blue and the SPR is shown in dark blue.

The os peroneum is of radiologic significance as its position can serve as an indicator of PLT injury. In cases of PLT injuries, there exists a spectrum of tendon and ossicle retractions, which may imply associated injuries to other anatomical structures such as the IPR and SPR [1,5]. Fig. 6 illustrates the anticipated anatomical migration of the os peroneum within a retracted PLT.

As the ossicle retracts proximally, indicative of PLT injury, it remains situated at the level of the lateral calcaneus if the IPR remains intact, or at least partially intact. Notably, fracture of the ossicle may occur in such cases [1,3]. It is imperative to differentiate fractures of this ossicle from bipartite or multipartite ossicles.

Further proximal retraction of the os peroneum between the IPR and SPR signifies injury to the IPR, while the SPR remains intact. The ossicle assumes a position either within or in close proximity to the SPR; radiographs demonstrate the ossicle distal to the lateral malleolus [4]. Finally, displacement of the ossicle proximally along the distal fibula signifies injury to the SPR, accompanied by additional PLT retraction.

These observations are indicative of progressively severe injuries to the PLT. MRI can aid in the diagnosis of PLT injuries with a characteristic empty, fluid-filled tendon sheath, and it can also help in assessment of the extent of the tendon tear [5].

As the function of PLT includes foot eversion and plantar flexion at the ankle, PLT injury can cause lateral foot and ankle pain and may lead to ankle instability [2]. This might predispose the patient to traumatic injuries, such as falls and missteps, which in this case led to the fifth metatarsal fracture. A fifth metatarsal base fracture is a common injury; rarely it can be seen in combination with a fracture of the os peroneum [6]. Radiographic follow-up is recommended when os peroneum migration is suspected in order to evaluate for progression of tendon injury and assess for associated fractures.

Given the potential implications in the management of lateral plantar foot pain, it is imperative for radiologists to scrutinize the appearance and position of the os peroneum on imaging studies. Pathology of the os peroneum, including fracture, enlargement or entrapment at the cuboid tunnel is highly associated with PLT tears [7,8]. Such attention to detail aids in accurate diagnosis, precise characterization, and subsequent management of patients presenting with lateral foot pain.

## Conclusion

The position of the os peroneum can serve as an indicator of injuries to the PLT and its associated structures. Radiographs can effectively detect malposition of the os peroneum. For patients with recurrent foot and ankle pain, it is imperative for radiologists to carefully examine the appearance and position of the os peroneum on imaging studies.

## Patient consent

Informed consent for this case was obtained from the patient.
